# Genomics-Assisted Improvement of Super High-Yield Hybrid Rice Variety “Super 1000” for Resistance to Bacterial Blight and Blast Diseases

**DOI:** 10.3389/fpls.2022.881244

**Published:** 2022-05-20

**Authors:** Zhizhou He, Yeyun Xin, Chunlian Wang, Hanshu Yang, Zhi Xu, Jihua Cheng, Zhouwei Li, Changrong Ye, Hexing Yin, Zhenyu Xie, Nan Jiang, Jing Huang, Jinhua Xiao, Bingchuan Tian, Yan Liang, Kaijun Zhao, Junhua Peng

**Affiliations:** ^1^Huazhi Bio-Tech Co., Ltd., Changsha, China; ^2^Tropical Crops Genetic Resources Institute, Chinese Academy of Tropical Agricultural Sciences, Haikou, China; ^3^China National Hybrid Rice Research and Development Center, Changsha, China; ^4^National Key Facility for Crop Gene Resources and Genetic Improvement (NFCRI), Institute of Crop Science, Chinese Academy of Agriculture Sciences (CAAS), Beijing, China; ^5^Key Laboratory of Southern Rice Innovation and Improvement, Ministry of Agriculture and Rural Affairs, Hunan Engineering Laboratory of Disease and Pest Resistant Rice Breeding, Yuan Longping High-Tech Agriculture Company Ltd., Changsha, China; ^6^State Key Laboratory of Rice Biology, China National Rice Research Institute, Hangzhou, China

**Keywords:** hybrid rice, Super 1000, R900, bacterial blight and blast resistance, genomics-assisted selection

## Abstract

The two-line rice hybrid “Super 1000” (GX24S × R900) represents a major landmark achievement of breeding for super-hybrid rice in China. However, both male parent R900 and hybrid “Super 1000” have an obvious defect of high susceptibility to rice bacterial blight (BB) and blast. Thus, improving disease resistance and maintaining the original high-yield capacity are essential for the sustainable application of “Super 1000.” In this study, the application of closely linked single-nucleotide polymorphism (SNP) markers for foreground selection of dominant resistance gene loci together with genome-wide SNP markers for the background selection rapidly improved the disease resistance of R900 without disturbing its high-yield capacity. A series of improved R900 lines (iR900, in BC_2_Fn and BC_3_Fn generations) were developed to stack resistance genes (*Xa23*+*Pi9, Xa23*+*Pi1*+*Pi2/9*) by marker-assisted backcrossing and field selection for phenotypes, and further crossed with the female line GX24S to obtain improved hybrid variety Super 1000 (iS1000). The genetic backgrounds of iS1000 and “Super 1000” were profiled by using a 56 K SNP-Chip, and results showed that they shared 98.76% of similarity. Meanwhile, evaluation of the field disease resistance showed that the iR900 lines and iS1000 hybrids possess significantly enhanced resistance to both BB and rice blast. Resistance spectrum assays revealed that the iR900 lines and their derived hybrids exhibited high-level resistance to 28 *Xoo* strains tested, and enhanced resistance to leaf blast at the seedling stage when infected with 38 *Magnaporthe oryzae* isolates. Between 2019 and 2020, the multi-location field trials across the middle and lower reaches of the Yangtze River were launched and showed that the iS1000 slightly out-yielded than the original variety. In a large-scale demonstration site (6.73 ha, Yunnan, China), the iS1000 achieved 17.06 t/hm^2^ of yield in 2019. Moreover, the high similarity was observed in main agronomic traits and grain quality when comparing the improved lines/hybrids to original ones (iR900 vs. R900, iS1000 vs. S1000). This work presented a typical genomics-assisted breeding strategy and practice, which involves in directional introgression and rapid stack of multiple disease resistance genes, endowing the super-high-yield hybrid rice variety with holistic disease resistance but without yield penalty.

## Introduction

Hybrid rice with high yield has greatly contributed to the increase in food production over the past half-century (Ma and Yuan, 2015). To better harness the yield potential, China has launched the “super-hybrid rice breeding program” in 1996. Since 2001, several pioneer hybrid rice combinations have been developed, and these super-hybrids roughly yielded 8.3 t/ha in commercial production. In 2014, a super-hybrid rice variety “Y Liangyou 900” marked a milestone with a yield of 15.4 t/ha (Yuan, 2018). Recently, another super-hybrid rice variety “Super 1000” constantly yielded 16 t/ha, and set the world record of rice yield (17.28 t/ha) in Yunnan province of China in 2018 (He et al., 2020). Efforts have still been made for a sustainable increase in rice yield. However, a foreseeable challenge is that the high yield potential of hybrid rice varieties is frequently threatened by various stresses. Therefore, it is imperative to rapidly develop stress-resilient rice varieties to ensure food security (Springer and Schmitz, 2017).

Rice bacterial blight (BB) and rice blast caused by *Xanthomonas oryzae* pv. *Oryzae (Xoo)* and *Magnaporthe oryzae*, respectively, are the two most prevalent and destructive diseases in global rice production (Wing et al., 2018; Raina et al., 2019; Samal et al., 2019). The rice blast affects crops in more than 80 countries, and can lead to yield losses by as high as 50% (Dayton, 2014). Similarly, rice BB affects millions of hectares of rice annually, with an estimated crop loss of as high as 75% (Zhao and Zhang, 2021). Super-high-yield hybrid rice breeding has made great progress in boosting rice yield in China, but its production is seriously threatened by rice blast and BB attributed to extensive use of a few non-resistant parents over the past years. Tremendous efforts have been devoted to characterizing the genetic diversity of rice germplasm resources to discover resistance genes. To date, at least 17 genes for BB resistance (Xu et al., 2022) and 25 genes for rice blast resistance have been cloned and are available for breeding purposes (Wing et al., 2018). Among these genes, the BB resistance gene *Xa23* is a new executor R gene located on the long arm of rice chromosome 11, and confers a dominant and extremely broad-spectrum of resistance against BB at all growth stages. The BB resistance gene *Xa23* is not expressed under normal conditions but can be activated immediately in the face of *Xoo*'s attack, and trigger the strong hypersensitive response at the infection site, thereby limiting the expansion of the pathogen, resulting in a high-level BB resistance (Wang et al., 2015).

For rice blast, the resistance gene *Pi2* confers dominant and broad-spectrum resistance to diverse *M. oryzae* isolates, and was mapped on the short arm close to the centromere of chromosome 6, at a locus carrying at least six RB resistance genes (*Pi2, Pi9, Pigm, Piz-t, Piz*, and *Pi50*; Liu et al., 2002; Hayashi et al., 2004; Deng et al., 2006; Zhu et al., 2012). In addition, *Pi1* is an allele at the *Pik* locus located at the end of the long arm of rice chromosome 11, and also confers dominant and durable resistance to a broad spectrum of rice blast isolates (Yu et al., 1996; Hua et al., 2012). Among those resistance genes, *Xa23, Pi1, Pi2/Pi9* have been widely used in the marker-assisted hybrid rice breeding programs due to their dominant effect and broad-spectrum resistance.

The development of rice varieties with durable and broad-spectrum resistance is an effective, economical, and environmentally sound way to prevent diseases (Wang and Valent, 2017). However, the conventional breeding approach takes many years to incorporate resistance into a susceptible (S) rice variety. Furthermore, resistance genes are sometimes associated with yield penalty because of linkage drag or unknown genetic background effects (Deng et al., 2017; Nelson et al., 2018). Genomic breeding technology is now widely applied to improve breeding efficiency by using foreground and background selection with the assistance of molecular markers (Bailey-Serres et al., 2019). Foreground selection enables the precise selection of individuals carrying the desirable target genes with the help of the marker information during the breeding process (Bai et al., 2006). The background selection devotes to accelerate the genomic recovery of the recipient parent genotype (RPG) by the assistance of markers across the whole genome (Hillel et al., 1990; Hospital et al., 1992; Xu et al., 2012). Computer simulations demonstrated that use of molecular markers for the background selection can accelerate recovery of the RPG by two or three generations (Tanksley et al., 1989). Large numbers of informative markers are available for use in the background selections, and these include single-nucleotide polymorphisms (SNP) that can be assayed in high-throughput genotyping arrays (Bevan et al., 2017). A lot of high-throughput genotyping platforms are established in recent years, and SNP markers are more efficient in terms of time and cost compared with other markers when a large number of markers are required for the background selection (Zhang et al., 2017; Wing et al., 2018). Breeders can use genome-wide markers to eliminate 70–80% of individuals in breeding generations without having to invest in laborious field testing (McCouch et al., 2013). Further, the combination of conventional breeding and genomic technologies, including foreground, background, and phenotypic selections, provides powerful options for increasing environmental resilience and productivity (Jez Joseph et al., 2016). The selection of plant traits by using these tools can significantly accelerate market entry of new varieties.

The hybrid rice restorer line R900 is a major innovative achievement of super-high-yield rice breeding because “Super 1000,” a combination derived from R900, has set the high-yield records in many places in recent years. However, R900 and “Super 1000” are highly susceptible (HS) to BB and rice blast, which not only exposed a production risk but also limited their application in a large scale. We here demonstrate genomics-assisted rapid and directional improvement of R900 and the derived hybrid variety Super 1000 for broad-spectrum resistance to both BB and rice blast.

## Materials and Methods

### Plant Materials

Plant materials used in this study include Super 1000 (a two-line-hybrid rice combination with super-high yield in China), R900 (the restorer line of Super 1000), GX24S (the PTGMS line of Super 1000), and resistance gene donors (HZ02455 containing *Xa23* + *Pi9* and HZ02411 containing *Pi1* + *Pi2*). The rice varieties JG30 and CO39 were used as S controls, while CBB23 and Gumei4 were used as resistant (R) controls.

### Population Development and Breeding Selection Procedure

**Figure 1:**
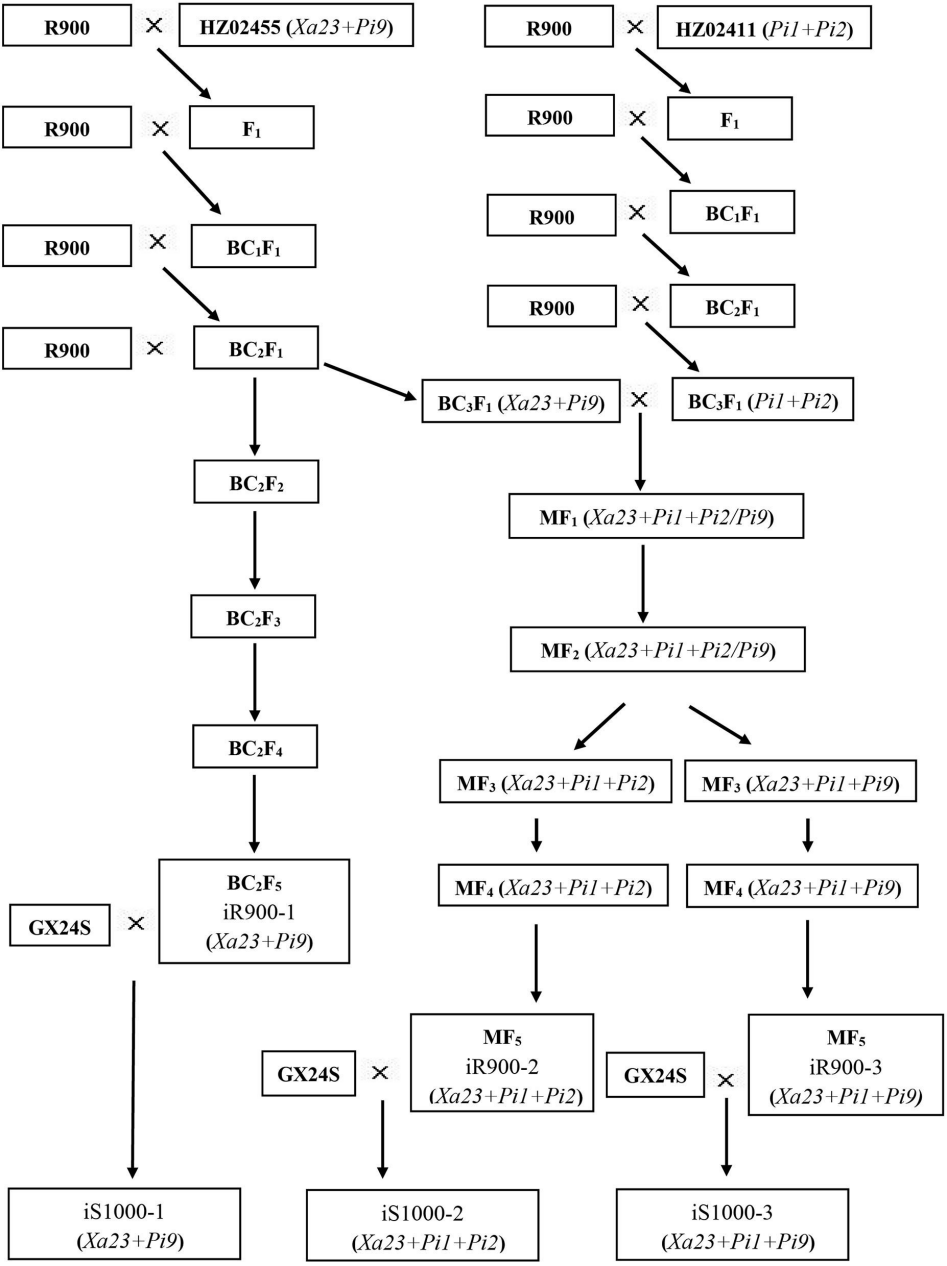
The scheme for development of the iR900 lines and iS1000 hybrids.

The improved R900 (iR900) lines and improved Super 1000 (iS1000) hybrids were developed by marker-assisted backcrossing as described in Figure 1. The hybrid rice restorer line R900 was crossed with resistance donors and the F_1_ plants were backcrossed with the recurrent parent R900. In each backcross generation, all individual plants were genotyped for foreground selection, and the plants containing the target genes were genotyped for the background selection. To pyramid the three resistance genes, double-cross F_1_ population was generated for marker-assisted selection. Finally, a series of improved lines iR900-1 (*Xa23* + *Pi9*), iR900-2 (*Xa23* + *Pi1* + *Pi2*), and iR900-3 (*Xa23* + *Pi1* + *Pi9*) were developed. These improved lines were then crossed with GX24S, the PTGMS line of Super 1000, to obtain the iS1000 hybrids iS1000-1 (*Xa23* + *Pi9*), iS1000-2 (*Xa23* + *Pi1* + *Pi2*), and iS1000-3 (*Xa23* + *Pi1* + *Pi9*) for disease-resistance and agronomic evaluations.

### Genomics-Assisted Foreground and Background Selection

Closely linked SNP markers, which are located upstream and downstream of the target genes (*Xa23, Pi1, Pi2/Pi9*), were used to track the target genes for foreground selection. By screening the whole genome SNP markers of recipient parent and donor parents, a total of 120 polymorphic SNP markers evenly distributed on each chromosome were selected for the background selection.

The fresh young leaves of 3-leaf seedlings were collected and placed in a 96-well plate, DNA was extracted by using Cetyltrimethylammonium Bromide (CTAB) method (Doyle and Doyle, 1987) on the TECAN Freedom EVO platform (Lifescience). Kompetitive allele-specific PCR (KASP) primers for each SNP were designed using BatchPrimer 3 (You et al., 2008). Genotyping was carried out on the Douglas Array Type and Nexar ultra-high-throughput genotyping platform (Douglas) following the user's manuals.

The background recovery rate was calculated as in the following:


$$\begin{array}{l} \text{Background recovery rate}=\left({\text{a}}^{*}2+\text{b}\right)/\left[\left(\text{a}+\text{b}+\text{c}\right)\times 2\right]\times \;100 \end{array}$$

Where *a* is the number of homozygous genotype loci identical to the donor (for example, AA), *b* is the number of heterozygous genotype loci (Aa), and *c* is the number of homozygous genotype loci (aa) identical to the receptor.

### Genetic Background Recovery Profiling by RICE 56K Gene Chip

The DNA samples were prepared using CTAB method for genotyping with a high-density Affymetrix 56 K SNP Chip. The rice 56K SNP chip contains 56,897 SNP markers selected from the dataset of whole-genome resequencing of 3,024 rice core germplasm from 89 countries or regions around the world (Li et al., 2014). The DNA amplification, fragmentation, chip hybridization, DNA ligation, and signal amplification were performed using the Affymetrix Axiom^®^ 2.0 Assay Manual Target Prep Protocol QRC. Staining and scanning were performed on the GeneTitan^®^ Multi-Channel Instrument (ThermoFisher) according to the manufacturer's procedure. Monomorphic markers, and/or showing unclear SNPs were excluded from the analysis. The filtered genotypic data was used for the background analysis.

### Evaluation of BB Resistance

The hybrid rice restorer line R900 and the iR900-1(harboring *Xa23*), Super 1000 (S1000), and the iS1000-1 (harboring *Xa23*) were planted in the field (Sanya, Hainan), during the dry season in 2019–2020 for BB resistance evaluation. The *indica* rice variety JG30 and its near-isogenic R line CBB23 (harboring *Xa23*) are used as S and R controls, respectively. Twenty-eight *Xoo* strains (Table 1) from different countries used in this study are stocks in Kaijun Zhao's laboratory. The *Xoo* cells were cultured in a PPS medium [ferv-filtering juice of 300-g potato, 5-g peptone, 15-g sucrose, 2-g Na_2_HPO_4_·12H_2_O, and 0.5g Ca (NO_3_)_2_·4H_2_O] at 28°C for 48 h. The bacterial inoculum was prepared by suspending bacterial culture in sterile, distilled water at an optical density of 1.0 (OD600). The bacterial suspensions were used for inoculation on fully expanded rice leaves at the booting stage. For each rice genotype, three plants were inoculated by the leaf-clipping method (Kauffman et al., 1973). For each plant, 3–5 fully expanded leaves were inoculated. The disease symptom was scored by lesion length (cm) and photographs were taken 2 weeks post-inoculation. The leaves with lesion length shorter than 1.0, 1.1–2.5, and longer than 2.5 were classified as highly resistant (HR), R and S, respectively.

**Table 1:** Resistance assessment of rice genotypes against 28 *Xoo* strains^a^.

***Xoo* strain**	**Origin of *Xoo* strain**	**Rice genotype**					
		**JG30**	**R900**	**S1000**	**iR900-1**	**iS1000-1**	**CBB23**
HLJ-72	China	6.47	6.93	7.53	2.00	0.10	0.67
HB17	China	7.20	5.93	5.50	1.47	0.47	1.27
NX42	China	10.07	5.90	6.57	3.87	0.90	1.17
ZHE173	China	3.97	5.50	2.20	1.10	0.40	0.83
GD1358	China	8.10	6.87	4.27	2.43	0.50	0.87
LN57	China	7.33	5.77	6.20	1.53	0.37	0.83
JS49-6	China	4.63	6.37	6.73	2.50	0.53	1.50
PXO61	Philippines	6.10	4.17	4.40	0.67	0.10	0.50
PXO86	Philippines	4.00	5.70	2.93	1.93	0.73	0.67
PXO71	Philippines	6.10	5.03	4.23	1.33	2.30	0.67
PXO112	Philippines	7.00	7.10	8.47	0.97	0.25	0.53
PXO99	Philippines	8.00	6.80	7.27	1.03	0.10	0.10
PXO280	Philippines	7.10	3.00	4.40	0.23	0.25	0.53
PXO339	Philippines	4.80	2.93	3.50	1.63	0.10	0.40
PXO341	Philippines	11.43	2.87	6.17	2.17	0.45	0.43
T7174	Japan	8.50	10.80	12.10	0.10	0.10	0.10
T7147	Japan	4.93	3.23	3.63	0.80	0.10	1.17
T7133	Japan	4.97	4.87	4.87	2.73	2.33	0.47
KXO19	Korea	12.80	6.13	7.60	2.50	0.10	0.30
KXO85	Korea	10.17	8.03	8.20	1.80	0.17	0.90
KXO576	Korea	8.13	5.17	3.23	0.40	0.30	0.10
PP8511	Bangladesh	7.20	4.47	8.37	1.33	0.45	0.83
GX15-4	China	11.77	7.47	7.57	1.80	0.40	0.80
YUN17-3	China	10.90	10.53	9.03	2.00	0.60	0.77
YUN17-8	China	10.73	9.50	11.87	2.57	0.83	0.67
YUN18-2-2	China	11.17	5.57	4.63	2.30	0.97	2.43
YUN18-16	China	9.57	8.80	6.30	0.93	1.10	0.80
LN44	China	11.37	8.77	7.43	1.73	3.33	1.97
Mean (cm)		8.02aA	6.22bA	6.26bA	1.64cB	0.65cB	0.80cB

a *The inoculation assays were performed during 2019-2020 in Sanya Experimental Station of CAAS, Hainan Province, China. The data in the table is mean BB lesion length (cm). Lesion length for each rice line against each Xoo strain was the mean of three leaves, measured at 14 days after inoculation. For the mean lesion length of 28 Xoo strains (last row in the table), the values followed by a common letter are not significantly different as determined by the least-significant difference (LSD) test at p < 0.01 (small letter) or p < 0.001 (capital letter), respectively*.

### Evaluation of Rice Blast Resistance

R900 and iR900-1 (harboring *Pi9*), iR900-2 (harboring *Pi1* and *Pi2*), iR900-3 (harboring *Pi1* and *Pi9*), were planted in the State Key Laboratory of Rice Biology, China National Rice Research Institute (Hangzhou, Zhejiang) during 2019–2020 for blast resistance evaluation at seedling stage by spraying inoculation (Yu et al., 2013). The *indica* rice variety CO39 was used as the S control. For each rice genotype, at least 15 plants at the 3–4-leaf stage were inoculated by spraying spores of *M. Oryzae*. The disease levels are scored following a standard 0–9 rating evaluation system (IRRI, 1996; Latif et al., 2011), wherein scores 0–1, 2, 3, 4-6, 7, and 8–9 represent HR, R, moderately resistant (MR), moderately susceptible (MS), S, and HS, respectively. A total of 38 *M. Oryzae* isolates (Table 2) were used for spraying inoculation, and disease evaluation was performed in triplicates.

**Table 2:** The detailed information of the *M. Oryzae* isolates and the disease reaction evaluated using a 0–9 rating system.

**Isolate code**	**Place of origin**	**Rice genotype**				
		**CO39**	**R900**	**R900-*Pi9***	**R900-*Pi1 + Pi9***	**R900-*Pi1 + Pi2***
19-765-1-4	Zhejiang, China	7	5	1	1	3
16-755-1-4	Zhejiang, China	9	0	0	7	0
19-763-7-2	Zhejiang, China	7	7	1	1	3
19-763-7-1	Zhejiang, China	9	1	1	3	3
19-765-1-3	Zhejiang, China	9	0	0	0	3
19-765-1-2	Zhejiang, China	9	5	7	5	3
16-755-2-4	Zhejiang, China	7	0	0	0	3
18-550-2-12	Hunan, China	0	1	3	0	1
2016CH2	Hunan, China	0	0	5	0	0
2016CH48	Hunan, China	5	7	0	5	5
2016CH47	Hunan, China	0	3	3	5	0
2016CH1	Hunan, China	9	0	0	0	0
2016CH50	Hunan, China	9	0	7	3	5
2016CH51	Hunan, China	9	5	5	0	0
2016CH52	Hunan, China	9	1	3	3	1
20-767	Zhejiang, China	9	0	3	1	3
2016CH49	Hunan, China	9	9	7	5	5
2016CH8	Hunan, China	9	3	5	5	5
2016CH7	Hunan, China	7	5	3	0	0
2016GD-1	Guangdong, China	5	3	3	3	1
2016CH-5	Hunan, China	9	3	3	0	3
2016ZY10	Unknown	3	9	7	9	9
2016ZY-1	Unknown	9	5	3	3	5
2016CH6	Hunan, China	9	9	5	7	3
2016GD2	Guangdong, China	9	5	0	3	3
18-162-7-1	Jiangxi, China	9	0	7	3	5
18-162-1-2	Jiangxi, China	0	0	0	0	0
195-2-2	Unknown	5	1	0	1	0
Z36-2	Unknown	7	9	0	1	0
RB10	Guangdong, China	9	9	5	3	3
18-162-5-1	Jiangxi, China	7	0	0	0	0
Z36-1	Unknown	7	0	0	0	0
18-162-5-4	Jiangxi, China	0	7	9	5	5
P06-6	Philippines	9	7	9	5	3
WJ2359	Wujin, China	9	5	7	5	7
2016ZY-7	Unknown	7	5	1	3	5
2016ZY-7	Unknown	9	0	0	0	0
RB14	Unknown	7	5	9	3	5

Leaf and neck blast resistances were evaluated under natural blast nurseries of two locations (Changsha and Jiangyong, Hunan) in 2020 and 2021. Eighteen plants were planted in the disease nursery for R900, the iR900 lines, Super 1000, and the iS1000 hybrids. The HS cultivar CO39 was sown on the plot borders. The field screening of resistance was performed by using a randomized block design with three replications. Additionally, the seedling was assessed for blast resistance by artificial inoculation with a mix of four representative isolates collected from Qionglai and Pujiang of Sichuan Province, Jiangyong and Changsha of Hunan Province, following the method described by Liu et al. (2019) with a minor modification, conidia were suspended to a concentration of 1 × 10^5^ spores per ml in 0.1% (w/v) TWEEN-20. A volume of 4 ml suspension was sprayed on 14-day-old rice seedlings. Inoculated plants were kept in a growth chamber at 28°C with 90% humidity and in the dark for the first 24 h, followed by a 14/10-h light/dark cycle. The disease symptom was assessed 1 week after inoculation according to the evaluation system above (IRRI, 1996; Latif et al., 2011).

### Evaluation of Yield, Main Agronomic Traits, and Grain Quality of the iR900 Lines and iS1000 Hybrids

The iR900-1 lines (containing resistance genes *Xa23* + *Pi9*) and the recurrent parent R900 were planted with one replication in the summer season in 2019, at Changsha, Hunan Province. Each plot comprised of five rows with eight plants per row at a planting density of 20 cm between plants and rows. Based on field observation, the five best-improved lines were selected. Ten plants with days to heading recorded in the middle of the central rows in each plot were sampled for measurements of agronomic traits, including plant height, panicle number, panicle length, spikelets per panicle, filled-grain percentage, 1,000-grain weight, and yield per plant. The agronomic traits were measured according to the standard evaluation system for rice (IRRI, 1996).

The iS1000 containing *Xa23* + *Pi9* (iS1000-1) and the original Super 1000 (S1000) were planted in the summer season in 2019 at seven locations as described in He et al. (2020). A large-scale (6.73 ha) field demonstration under super-high-yielding cultivation practices were also performed for the iS1000-1 in Yunnan Province in 2019, The yield was evaluated by an expert group in accordance with the yield measurement method for super-rice of the Ministry of Agriculture and Rural Affairs of China. The iS1000-2 containing *Xa23* + *Pi1* + *Pi2*, iS1000-3 containing *Xa23* + *Pi1* + *Pi9*, and the original Super 1000 were planted in the summer season in 2020 at five locations in the middle and lower reaches of the Yangtze River using the method described by the China National Crop Variety Approval Committee. Yield per plot, seven main agronomic traits, and five grain quality traits were analyzed. To compare yield and yield components under the disease-stressed environment, another trial was conducted for the iS1000-2 and the original Super 1000 in the occurrence of natural blast epidemic and artificially BB-inoculated in Changsha, Hunan Province. Yield per plot and four yield component traits were investigated. The *t*-test was performed to examine the statistical significance of differences in yield and agronomic traits by using Microsoft Office Excel software.

## Results

### Development of the iR900 Lines and iS1000 Hybrid Containing *Xa23* and *Pi9* Genes

All the foreground and the background SNP markers were used to genotype R900, HZ02455 (donor of *Xa23* + *Pi9*), and the F_1_ plants to confirm the polymorphism of the markers. The F_1_ plants were then backcrossed with R900. A total of 368 BC_1_F_1_ plants were genotyped using the foreground markers, and 93 plants with genes *Xa23* and *Pi9* were selected for the background examination using 115 SNP markers. The background analysis revealed that the background recovery rate ranged from 63.9 to 82.6% in these 93 individuals. Three plants with the highest background recovery rate were selected to further backcross with R900 to develop the BC_2_F_1_ population. For the BC_2_F_1_, a total of 550 plants were genotyped using the foreground markers, and 142 plants with genes *Xa23* and *Pi9* were selected for the background examination using 60 SNP markers. The background analysis revealed that the background recovery rate ranged from 83.3 to 96.1%. Three plants with the highest background recovery rate were selected to further backcross with R900. In the BC_3_F_1_ population, a total of 92 plants were genotyped using the foreground markers, and 19 plants with genes *Xa23* and *Pi9* were selected for the background examination using 16 SNP markers. The background analysis revealed that the background recovery rate ranged from 95.9 to 99.0%. In parallel, the BC_2_F_1_ plant selected was also selfed to get BC_2_F_2_. In the BC_2_F_2_ population, 207 plants with genes *Xa23* and *Pi9* were genotyped for background selection using 32 SNP markers. The background analysis revealed that the background recovery rate ranged from 93.9 to 98.8%. Among them, the plants with the highest background recovery rate, homozygous genotypes of *Xa23* and *Pi9* genes, and maximum phenotypic similarity to R900 were selected for continuous self-pollination till BC_2_F_5_. The best plants (iR900-1) from the BC_2_F_5_ population were crossed with the sterile line GX24S to produce an improved “Super 1000” combination (iS1000-1) which contains *Xa23* + *Pi9* genes (Figure 1).

**Figure 2:**
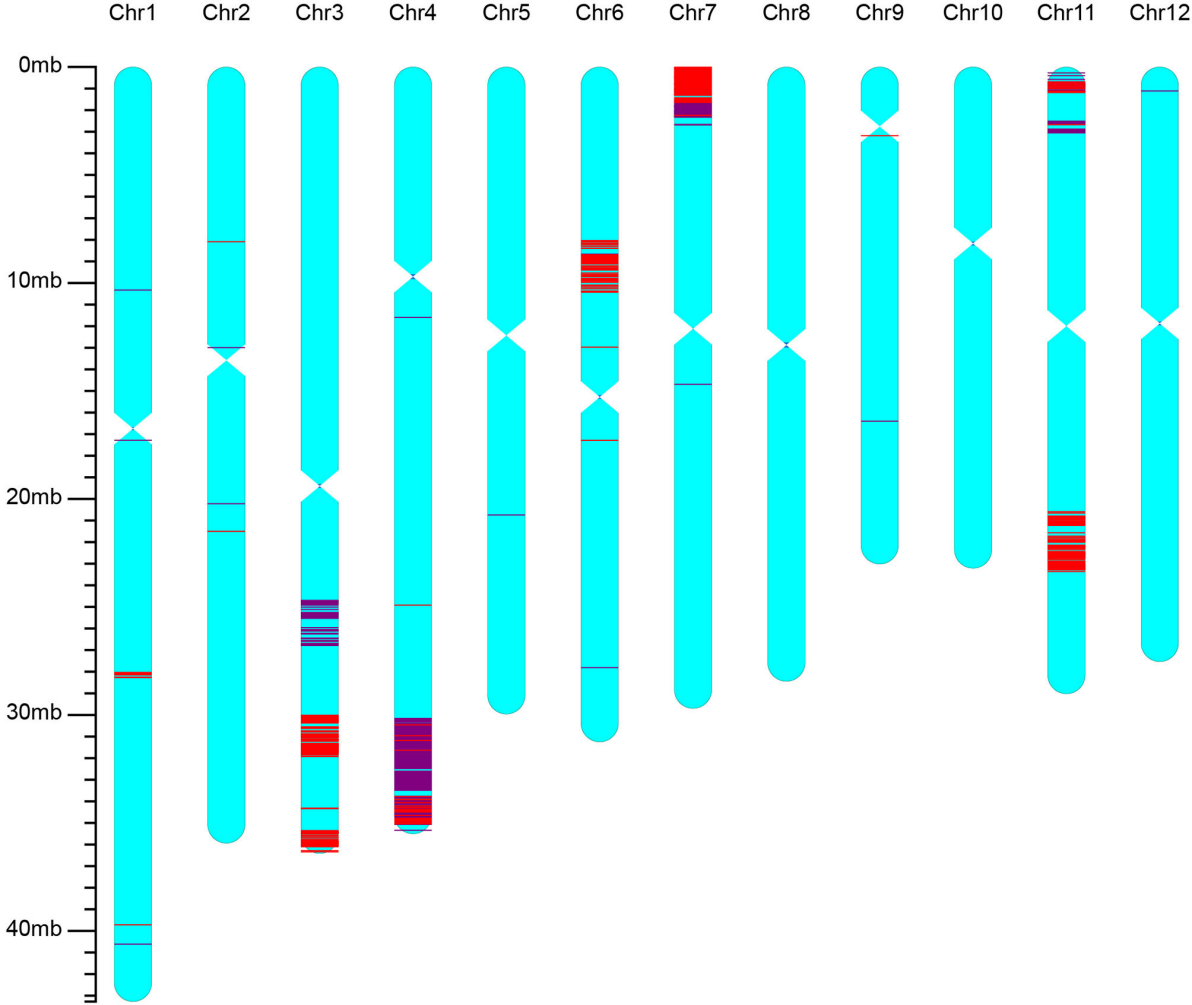
Genetic background of the iR900 line containing genes *Xa23* + *Pi9* revealed by using the rice 56K SNP chip. The red lines indicate the SNP loci with homozygous genotypes of the donor parent; the purple lines indicate the SNP loci with heterozygous genotypes.

**Figure 3:**
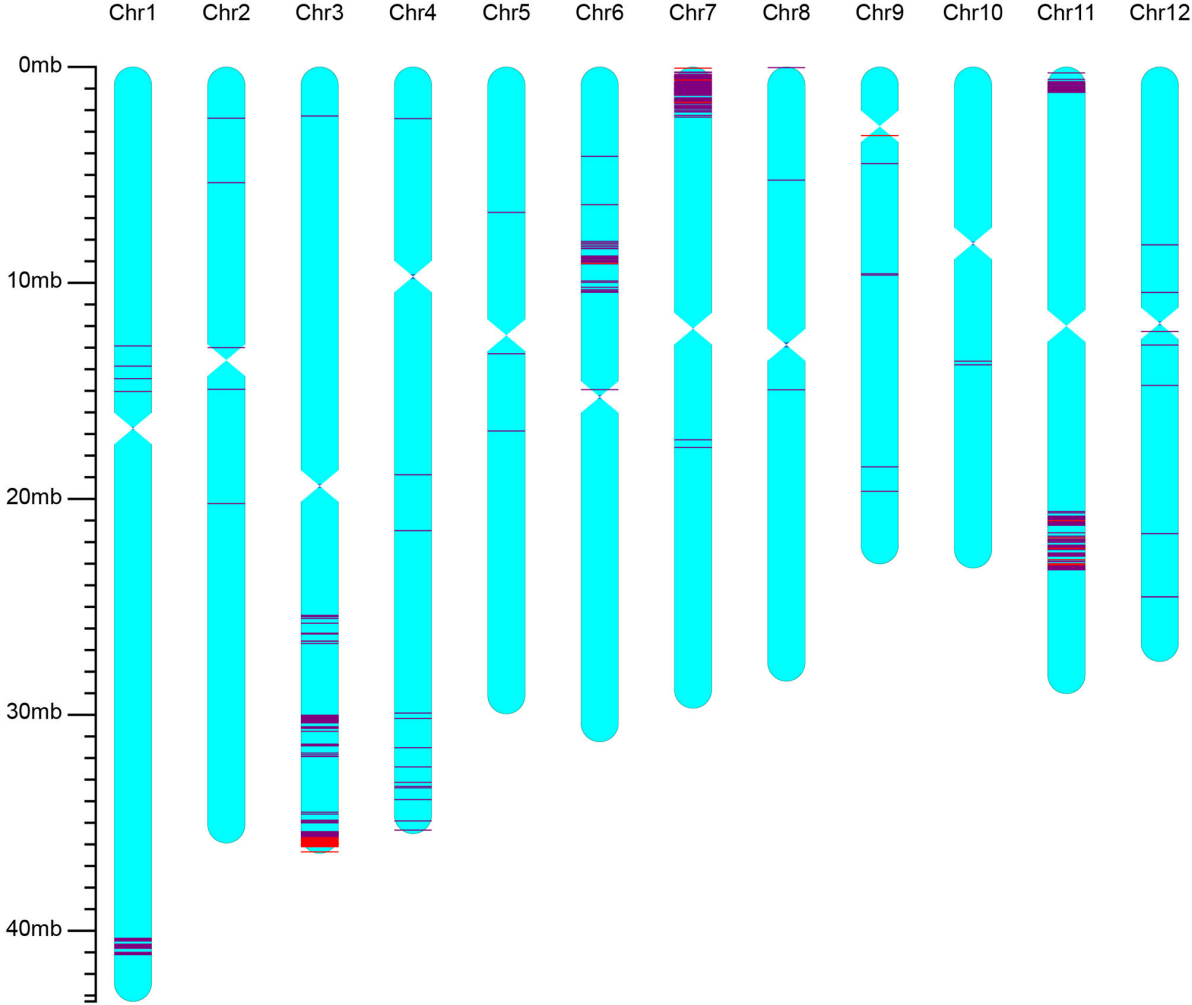
Genetic background of the iS1000 containing genes *Xa23* + *Pi9* revealed by using rice 56K SNP chip. The red lines indicate the SNP loci with homozygous genotypes of the donor parent, the purple lines indicate the SNP loci with heterozygous genotypes.

The best line selected from BC_2_F_3_ of the iR900 lines and its corresponding hybrid combination iSuper 1000 containing *Xa23* + *Pi9* genes were genotyped using the high-density 56K SNP chip to analyze the genetic background. It was found that the background recovery rate between the iR900-1 and R900 is 96.8% (Figure 2), and the genetic similarity between the improved Super 1000 (iS1000-1) and Super 1000 is 98.8% (Figure 3), slightly higher than that between the R900 and iR900 as expected.

### Development of the iR900 Lines and iS1000 Hybrid Containing Genes *Xa23 + Pi1* + *Pi2/ Pi9*

The F_1_ plants derived from the cross between R900 and HZ02411 (*Pi1* and *Pi2* donor) were genotyped using foreground SNP markers, and 10 F_1_ plants with target genes were backcrossed with R900. In the BC_1_F_1_ population, a total of 768 plants were genotyped using the foreground markers, and 186 plants with *Pi1* and *Pi2* genes were selected for the background genotyping using 111 SNP markers. The background analysis revealed that the background recovery rate ranged from 72.0 to 90.4%. Three plants with the highest background recovery rate were selected to backcross with R900. In the BC_2_F_1_ population, a total of 620 plants were genotyped using the foreground markers, and 145 plants with genes *Pi1* and *Pi2* were selected for the background genotyping using 32 SNP markers. The background recovery rate ranged from 87.9 to 95.4%, and two plants with the highest background recovery rate were selected to backcross with R900. In the BC_3_F_1_ population, a total of 94 plants were genotyped using foreground markers, and 21 plants with genes *Pi1* and *Pi2* were selected for the background genotyping using 11 SNP markers. The background analysis revealed that the background recovery rate ranged from 97.1 to 98.6%. To pyramid the genes *Xa23* + *Pi1* + *Pi2/Pi9* in the R900 genetic background, the best plants with *Xa23* and *Pi9* genes from the BC_3_F_1_ population of R900/HZ02455 were crossed with the best plants with genes *Pi1* and *Pi2* from BC_3_F_1_ population of R900/HZ02411 to produce multi-cross F_1_ (MF_1_) seeds. Based on the foreground selection of 72 MF_1_ plants, plants with *Xa23*+*Pi1*+*Pi2/Pi9* were selfed to generate MF_2_ seeds. Among 3,098 MF_2_ plants subjected to foreground screening, the plants with *Xa23* + *Pi1* + *Pi2/Pi9* and similar or superior agronomic traits to R900 were selfed to generate MF_3_ seeds. According to the foreground and field screening of the desired target traits and comprehensive agronomic traits, plants with homozygous genotypes of genes *Xa23* + *Pi1* + *Pi2* and *Xa23* + *Pi1* + *Pi9* and superior agronomic traits were selfed till MF_5_. The best plant in the MF_5_ population was crossed with the sterile line GX24S to produce two types of improved “Super 1000” hybrid combinations which contain genes *Xa23* + *Pi1* + *Pi2* (iS1000-2) and *Xa23* + *Pi1* + *Pi9* (iS1000-3) (Figure 1).

### Phenotypes of iR900-1 Line and iS1000-1 Hybrid for BB Resistance

**Figure 4:**
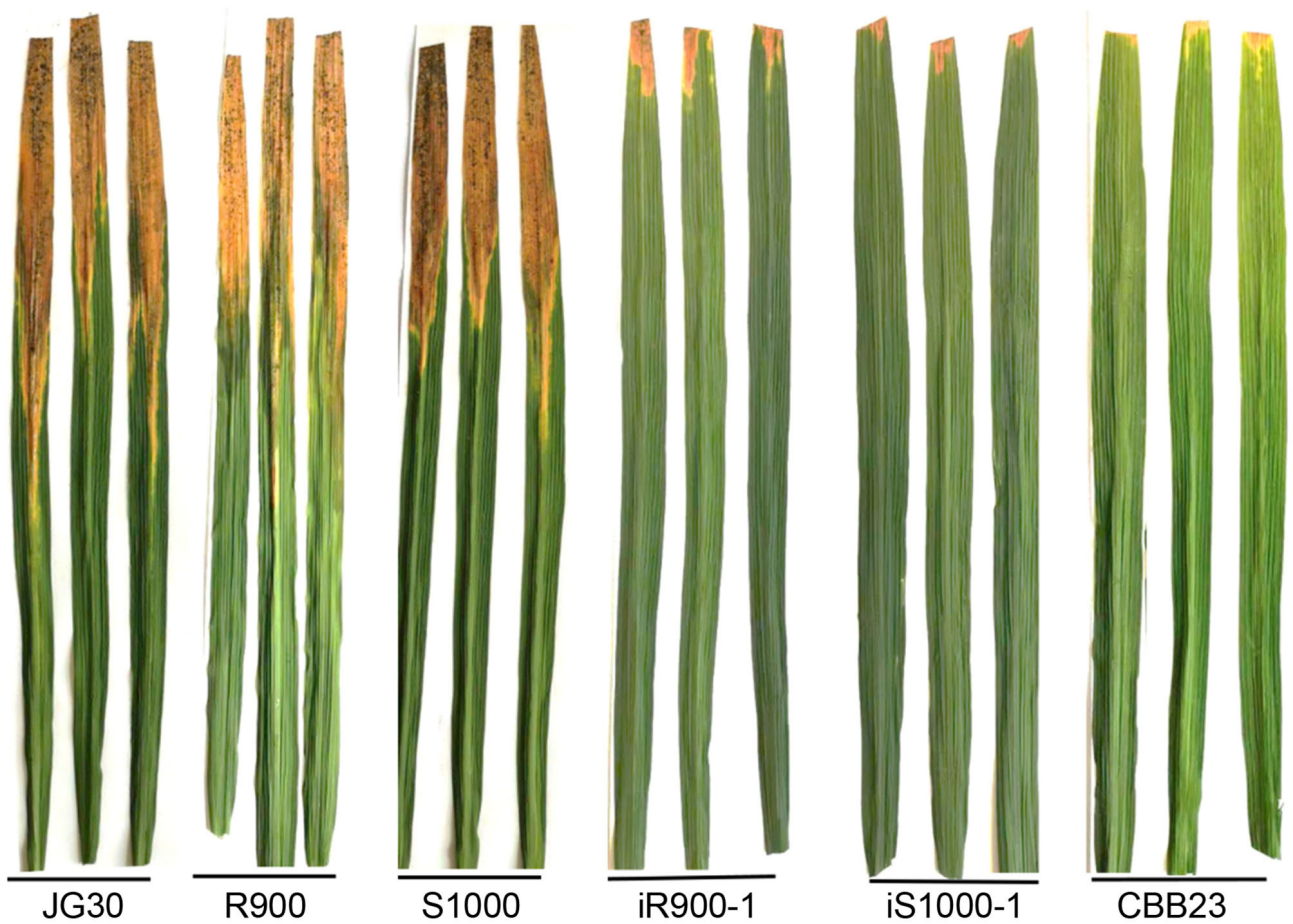
Assessment of BB resistance of 6 rice genotypes. The disease reactions of the rice genotypes, including S (JG30) and R (CBB23) checks, to the representative *Xoo* strain PXO99 inoculated using the leaf-clipping method. The R and S lesions 14 days after inoculation are shown by representative leaves.

A set of 28 individual *Xoo* strains were used to infect plant leaves for each of the genotypes to be examined. The degree of BB resistance is indexed by lesion length on leaves post-inoculation, and the shorter lesion length indicates higher resistance. The resistance assays showed that both R900 and S1000 are HS to BB. As showed in Table 1 and Figure 4, the lesion lengths of the recurrent parent R900 ranged from 2.87 cm (against PXO341) to 10.8 cm (against T7174) with the mean length of 6.22 cm (Table 1). Similarly, the lesion lengths of the hybrid Super 1000 (S1000) ranged from 2.2 cm (against ZHE173) to 12.1 cm (against T7174) with a mean length of 6.26 cm (Table 1). In contrast, the mean lesion lengths of the S control (JG30) and the R control (CBB23) are 8.02 and 0.80 cm, respectively (Table 1). These data also indicated that a specific rice genotype responds differently to different *Xoo* strains (Table 1).

As showed in Table 1 and Figure 4, weak symptom was found on the leaves of iR900-1 and iS1000-1 post-inoculation. The *Xoo* strains led to 1.64 cm (0.40–3.87 cm) of lesions in length for iR900-1, and 0.65 cm (0.10–0.90 cm) of lesions in length for iS1000-1 (Table 1, Figure 4), indicating that the resistance level of both iR900-1 and iS1000-1 is comparable to the R control CBB23 (Table 1, Figure 4). These results clearly demonstrated that introducing the *Xa23* gene enhanced the BB resistance remarkably in both parent iR900-1 and hybrid iS1000-1.

### Phenotypes of the iR900 Lines and iS1000 Hybrids for Rice Blast Resistance

**Figure 5:**
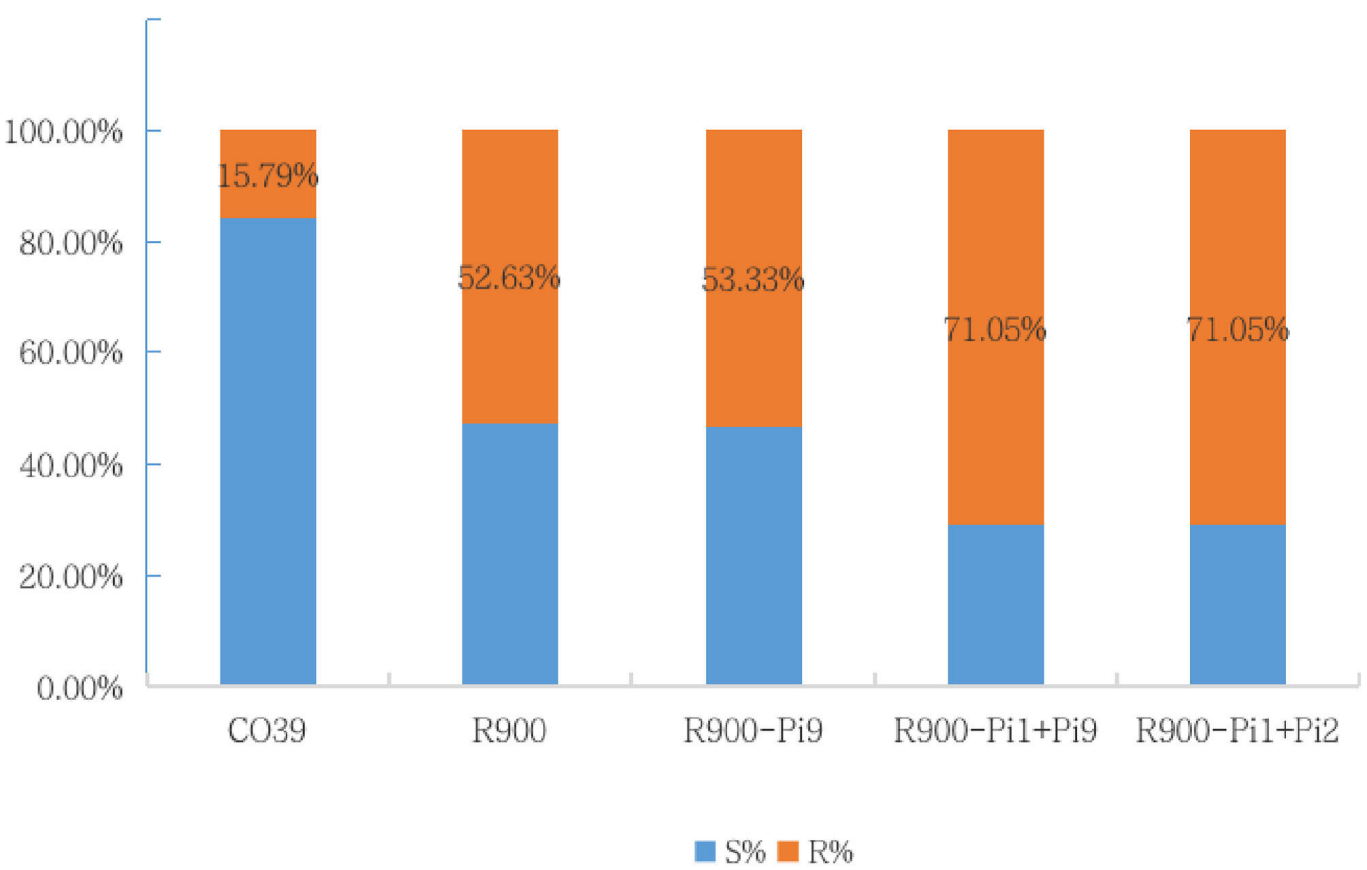
The resistance frequency/spectrum of five genotypes based on the disease reaction phenotype of spraying inoculation and disease evaluation at seedling stage.

The blast resistance reaction was evaluated at 7–10 days after spraying inoculation, using a 0–9 rating system (Table 2). The phenotypic results for infection of 38 *M. Oryzae* isolates were calculated for resistance frequency and a higher frequency represents a broader resistance spectrum. As showed in Figure 5, the resistance frequency of iR900-2 carrying *Pi1* + *Pi2*, and iR900-3 carrying *Pi1* + *Pi9* were 71.05%, which is clearly higher than that of R900 (52.63%), while the resistance frequency of S control CO39 is dramatically low (15.79%). These results verify that blast resistance is occurring in those lines with R genes. It is worth to note that the line iR900-1 carrying *Pi9* did not perform an improved resistance spectrum (resistance frequency: 53.33%) compared to R900 (resistance frequency: 52.63%), which may suggest that pyramiding of *Pi9* and *Pi1* is required for a broader blast resistance spectrum.

Blast resistance was also evaluated on rice panicle neck under field conditions (Changsha, Hunan) in 2020 and 2021. Panicle neck blast evaluation showed that the recurrent parent R900 was HS with 60% infection rate in Changsha, while the iR900 lines presented much higher resistance with 10–20% infection rate in the same nursery (Table 3). The iR900 lines with two R genes exhibited better panicle neck blast resistance than the line with only one R gene. For the hybrid combinations, Super 1000 was HS to blast in the 2 years at two locations, with leaf blast score ranging from 6 to 7 and panicle neck blast infection rate from 80 to 90% (Table 3). In contrast, the iS1000 hybrids exhibited resistance to blast with the highest score of 2 for leaf blast and 10% of the infection rate for panicle neck blast infection. The iS1000 with R genes *Pi1* + *Pi2* exhibited slightly better panicle neck blast resistance than the hybrid with R genes *Pi1* + *Pi9* in Changsha nursery in 2020 (Table 3).

**Table 3:** Performance of leaf and panicle neck blast resistances of the iR900 and iS1000 hybrids in natural blast nurseries.

**Location**	**Year**	**Line and hybrid**	**Leaf blast score**	**Panicle neck blast infection rate (%)**
Changsha, Hunan, China	2020	R900	-	60.0
		iR900-1 (*Xa23 + Pi9*)	-	20.0
		iR900-2 (*Xa23+Pi1+Pi2*)	-	10.0
		iR900-3 (*Xa23+Pi1+Pi9*)	-	10.0
		S1000	-	80.0
		iS1000-2 (*Xa23+Pi1+Pi2*)	-	6.7
		iS1000-3 (*Xa23+Pi1+Pi9*)	-	10.0
Jiangyong, Hunan, China	2020	S1000	-	86.6
		iS1000-2 (*Xa23+Pi1+Pi2*)	-	0
		iS1000-3 (*Xa23+Pi1+Pi9*)	-	0
Changsha, Hunan, China	2021	S1000	7	90.0
		iS1000-2 (*Xa23+Pi1+Pi2*)	0	8.3
Jiangyong, Hunan, China	2021	S1000	6	81.6
		iS1000-2 (*Xa23+Pi1+Pi2*)	2	1.4

**Figure 6:**
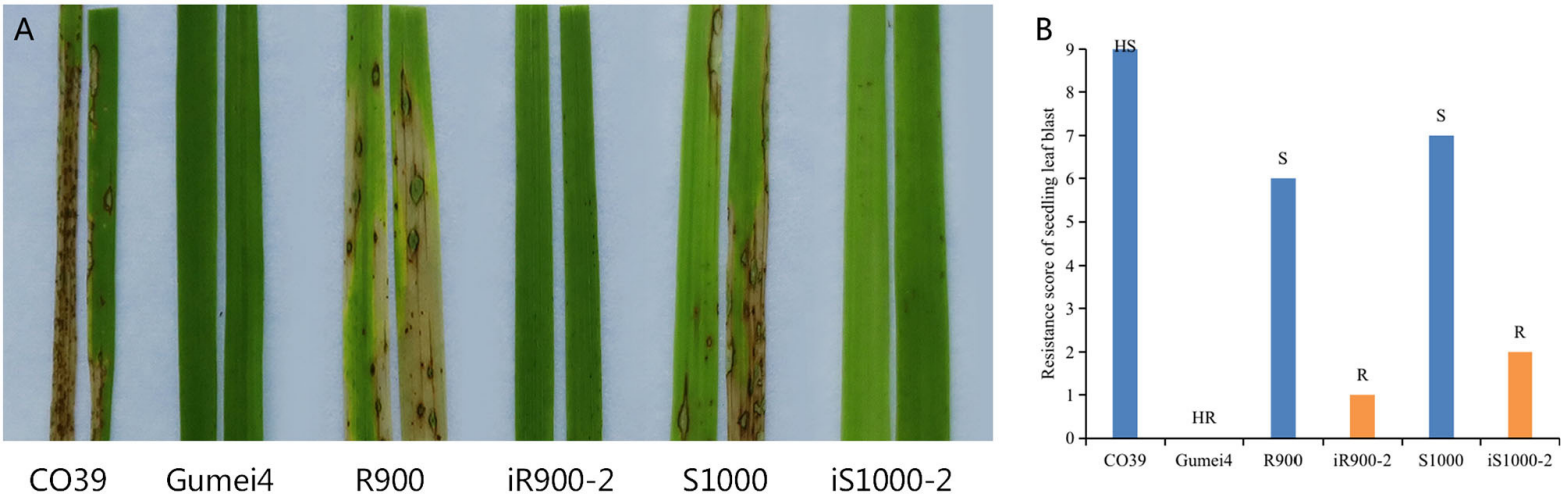
Evaluation of seedling blast resistance of six rice genotypes. **(A)** Disease reactions of the rice genotypes, including the S (CO39) and R (Gumei4) checks, to a mix of four isolates by using spraying inoculation. **(B)** Resistance score and resistance level. HR, highly resistant; R, resistant; MR, moderately resistant; S, susceptible; HS, highly susceptible.

Further, the seedling leaf blast resistance was tested by spray inoculation with a mix of four representative isolates. The checks CO39 and Gumei4 showed HS and HR to seedling leaf blast, with a score of 9 and 0, respectively (Figure 6). The original R900 and Super 1000 showed a resistance score of 6 (S) and 7 (S), while the iR900-2 and iS1000-2 presented a resistance score of 1 (R) and 2 (R) (Figure 6). In short, resistance to rice blast was effectively enhanced in the iR900 and its derived Super 1000 hybrid variety.

### Performance of Yield, Agronomic and Grain Quality Traits of the iR900 Lines and the iS1000 Hybrids

Five of the iR900-1 lines were evaluated for key agronomic traits as compared to the original R900. In general, no significant difference was observed between the iR900 lines and the original R900 for most of the agronomic traits (Table 4). Nevertheless, some differences were still detected for a few traits, including spikelet fertility, yield per plant, etc. For example, the spikelet fertility of the iR900-1-3 and iR900-1-4 was higher than that of the original R900 by 10 and 8.8%, respectively, and the yield per plant of the iR900-1-4 and iR900-1-5 was higher than that of the original R900 by 7.5 and 5.1 g, respectively. Furthermore, the panicle number of the iR900-1-4 outnumbered that of the original R900 by 0.9 per plant, and the 1,000-grain weight of the iR900-1-5 exceeded that of the original R900 by 2.3 g (Table 4). These results indicated that the main agronomic traits of the five selected lines are essentially identical, but the yield per plant and filled-grain percentage were superior to that of the recurrent parent, R900.

**Table 4:** The performance of yield and main agronomic traits of the iR900 lines containing genes *Xa23*+ *Pi9*.

**Line**	**Trait^a^**							
	**Yield (g/plant)**	**DTH(d)**	**PH (cm)**	**PN**	**PL (cm)**	**NGP**	**FGP (%)**	**GW (g)**
R900	23.1	98.7	108.6	3.8	24.7	358.2	71.9	24.0
iR900-1-1	26.0	98.3	109.5	4.5	24.2	315.9	74.8	25.5
iR900-1-2	27.0	99.6	107.2	4.6	23.4	338.0	73.4	24.0
iR900-1-3	27.8	100.2	108.2	3.9	23.7	348.6	81.9^**^	24.8
iR900-1-4	30.6^*^	99.3	111.0	4.7^*^	23.1	331.6	80.7^**^	24.7
iR900-1-5	28.2^*^	99.5	108.4	4.2	24.8	331.8	77.5	26.3^**^

a *DTH, days to heading; PH, plant height (cm); PN, panicle number/plant; PL, panicle length (cm); NGP, number of grains/panicle; FGP, filled-grain percentage (%); GW, 1,000-grain weight (g)*.

*,** *Significant difference with R900 at the 5 and 1% probability level, respectively*.

Large-scale multi-site field trials were launched in 2 consecutive years (2019 and 2020) to compare the iS1000-1 and the original Super 1000 for yield and some key agronomic traits. It was found that no significant difference was observed for yield and all of the tested agronomic traits in 2019 (He et al., 2020). The results in 2020 showed that the average yields of the two iS1000 hybrids were 9.9 t/ha and 9.6 t/ha which was higher than that of the original Super 1000 by 4.2 and 1.1%, respectively. We investigated seven agronomic traits (days to maturity, plant height, spikelets/panicle, filled-grain percentage, and 1,000-grain weight) and data were collected from five locations. Non-significant differences were found between the iS1000 hybrids and the original Super 1000 for the traits observed (Table 5).

**Table 5:** The performance of yield, main agronomic, and grain quality traits of the iS1000 hybrids.

**Hybrid**	**Trait^a^**												
	**Yield (t/hm^**2**^)**	**DTH**	**PH (cm)**	**PN**	**PL (cm)**	**NGP**	**FGP (%)**	**GW (g)**	**MRP (%)**	**L/W**	**CRP (%)**	**GC (mm)**	**AC (%)**
S1000	9.5	89	120.1	9.1	24.1	272.6	83.1	23.5	70.3	2.7	11.6	86.6	25.7
iS1000-2	9.9	88.6	121.2	10.2	24.2	275.8	83.4	23.5	70.4	2.7	12.9	88.3	26.9
iS1000-3	9.6	89.2	120.8	9.1	24.6	283.5	84	23.8	70.8	2.8	10.5	88.3	25.7

a *DTH, days to heading; PH, plant height (cm); PN, panicle number; PL, panicle length (cm); NGP, number of grains per panicle; FGP, filled-grain percentage (%); GW, 1,000-grain weight (g); MRP, milling rice percentage (%); L/W, grain length-width ratio; CRP, chalky rice percentage (%); GC, gel consistency (mm); AC, amylose content (%)*.

To profile grain quality, rice grains from three sites were collected and characterized for five important rice quality characteristics (milling rice percentage, grain length/width ratio, chalky rice percentage, gel consistency, and amylose content). It was shown that these traits were statistically identical between the iS1000 hybrid combinations and the original Super 1000 (Table 5).

Furthermore, yield potential was explored in a large-scale field demo trial with and without biotic stress for the improved and original Super 1000. The yield of a large-scale (6.73 ha) field demonstration (Yunnan, China) in 2019 showed that the iS1000 with resistance achieved a super-high yield of 17.1 t/hm^2^, which nearly reached a yield record of the original Super 1000 set in 2018.

Under the biotic stress conditions, the iS1000 also performed a constant high yield compared to the original Super 1000. Both original and two improved varieties were planted in Changsha for natural blast infection and BB inoculation. Under the serious blast outbreak conditions and BB stresses, the original Super 1000 had a yield loss of 26.6 and 31% compared with the two iS1000 hybrids, respectively. The yield components data showed that the filled-grain percentage of the original Super 1000 was declined by 24 and 18.2% compared with two iS1000 hybrids, which illustrated that the yield losses were caused by rice blast and BB epidemics (Table 6). In summary, all these results approved that the iR900 lines and the derived iS1000 hybrids maintained the key agronomic traits, some selected lines and their hybrids even performed higher yields to that of the non-improvement checks, the original R900 and Super 1000.

**Table 6:** Comparison of yield and yield components between the iS1000 and the original Super 1000 under disease stresses.

**Variety and environment**	**Trait^a^**				
	**Yield (t/hm^**2**^)**	**PN**	**NGP**	**FGP (%)**	**GW (g)**
S1000 under blast epidemic	7.6	8.9	262.8	60.9	23.1
iS1000-2 under blast epidemic	10.3	9.5	249.8	84.9	25.3
S1000 under BB artificial inoculation	5.9	7.2	261.1	47.7	23.6
iS1000-2 under BB artificial inoculation	8.6	7.2	280.9	65.9	24.2

a *PN, panicle number; NGP, number of grains per panicle; FGP, filled-grain percentage (%); GW, 1,000-grain weight (g)*.

## Discussion

### Fast Improvement Achieved by Genome-Wide Marker-Assisted Selections

Conventional breeding, an experience-based approach has played a great role to develop elite rice varieties in the past (Wing et al., 2018), but usually has a long breeding cycle and low efficiency. In general, it takes 8–10 years for a whole breeding cycle (Gao, 2021) and has considerable uncertainty for predicting results of the selected lines (Bevan et al., 2017). Alternatively, genomics technology based on genome information has been widely applied to improve breeding efficiency that is demonstrated in case of precise incorporation of target gene into elite varieties by the marker-assisted foreground and background selection. In this decade, the high-throughput and affordable genotyping platforms have enabled practical breeding to be performed in a rapid and precise manner that takes full advantage of genomic information (Herzog and Frisch, 2011; Springer and Schmitz, 2017; Wei et al., 2021). In this study, four dominant major resistance genes were introduced into the background of a super-hybrid-rice restorer line R900 by genomics-assisted selection. Two tightly linked SNP markers for each target gene and 120 polymorphic background SNP markers were applied for the genomic marker-assisted selections. In BC_1_F_1_ populations, the background recovery rate of the plants with target genes were 82.6 and 90.4%, much higher than the expected average of 75% in the first backcross generation. With backcross advanced to BC_3_F_1_, the background recovery rate of the target plants ranged from 95.9 to 99.0%, and the genetic background of recurrent parent R900 was rapidly recovered in the iR900 lines. In the recent reports, the background selection was mostly based on a few SSR markers which had low resolution and inadequate whole-genome coverage (Priyadarshi et al., 2018; Yang et al., 2019; Ramalingam et al., 2020). The SNP array with abundant markers is an efficient tool for the background analysis, which was almost 300 times more cost-effective in relative to SSR markers (Khanna et al., 2015). The analysis by high-density rice 56K SNP chip revealed that the selected line from BC_2_F_3_ of the iR900 has a background recovery rate of 96.8% to the recurrent parent R900. Therefore, genome-wide marker-assisted background selections can be performed to significantly reduce backcross generations in crop improvement (Hospital and Charcosset, 1997; Frisch et al., 1999; Frisch and Melchinger, 2001).

### Stacking Genes to Improve BB and Blast Resistance

The iR900 lines and the iS1000 hybrids with R genes clearly showed enhanced resistance against BB and rice blast diseases. The improved lines of R900 and the derived Super 1000 hybrid containing *Xa23* are HR to the 28 tested *Xoo* strains. These results implied that the *Xa23* gene has a dominant effect because both iR900 lines (*Xa23/Xa23*) and iS1000 hybrids (*Xa23/xa23*) can exhibit broad-spectrum resistance to BB isolates worldwide. It is known that the *Xa23* gene expresses in a dominant manner and confers strong resistance to BB with the broadest resistance spectrum that has been widely utilized in breeding programs (Zhou et al., 2009; Huang et al., 2012; Jiang et al., 2015). However, the other resistance genes, such as *Xa7* and *Xa21*, can be overcome in the recent years by the new virulent BB strains due to pathogenic variation and evolution (Zhang, 2009; Chen et al., 2011; Wang et al., 2014).

For resistance to blast at the seedling stage, the iR900 lines containing *Pi1* + *Pi2/Pi9* exhibited resistance to 71.05% of 38 *M. Oryzae* isolates under artificial inoculation, showing an obviously increased resistance frequency/spectrum compared with that of the original R900 (Figure 5). Furthermore, the field trial also showed that the iR900 lines presented a significantly increased resistance level for panicle neck blast, conferring the highest grade of resistance to leaf blast (score = 2) and lowest 10% of infection rate (10%) for panicle neck blast in the iS1000. These results revealed that it is feasible to stack multiple resistance genes for broad-spectrum resistance and increased resistance levels. It is more usual to use a single R gene in breeding for resistance. For instance, the *Pi9* gene was HR to 43 *M. oryzae* isolates from 13 countries (Liu et al., 2002); therefore, it has been widely employed in rice breeding programs (Ni et al., 2015; Luo et al., 2017). However, we found that integration of the *Pi9* gene alone into R900 is not applicable to broaden resistance spectrum and increase resistance level to blast. First, iR900-*Pi9* showed resistance to only 53.3% of the 38 *M. Oryzae* isolates tested, almost comparable to that of the recurrent parent R900. Second, both iR900-*Pi9* and iS1000-*Pi9* showed rather a high percentage of blast infection on panicle neck in the field nurseries, 20 and 95.6%, respectively (He et al., 2020), implying that a single *Pi9* gene is not effective for blast control in the background of R900 and its hybrid Super 1000. Owing to the dynamic and evolving nature of host-pathogen interactions, single-gene resistance is often easily overcome by evolving pathogen populations after a few years of cultivation. Resistance breeding is therefore an ongoing process, and resistance must be managed strategically (Nelson et al., 2018). While incorporating resistance gene into breeding strategies might improve the durability of resistance, there is an exigency for pyramiding multiple genes for attaining durable resistance (Hittalmani et al., 2000; Singh et al., 2012). As the results showed in our study, the resistance spectrum to blast is remarkably broadened by a combination of *Pi9* and *Pi1* (from 53.3 to 71.05%). In the field nursery, the iR900 lines with two blast genes also presented better panicle neck blast resistance than the single gene *Pi9* (Table 3). The stacking of several resistance genes with different recognition spectra and environmental optima into a single background is thus a credible strategy for achieving more durable disease resistance (Bailey-Serres et al., 2019).

### Improvement of Disease Resistance Without Yield Penalty

Crops' defense activation usually causes growth inhibition and yield reduction, which are referred to as trade-offs between growth and defense (Nelson et al., 2018). The genomics-assisted breeding strategy involves precise target genes identification and whole genomic background selection is helpful to avoid these problems. In our study, we developed large populations in BC_1_F_1_ and BC_2_F_1_ generations, more than 100 SNP markers covering the whole rice genome were used to analyze the genetic background. In the BC_2_F_1_ population, the highest background recovery rate reached over 95%. The field trial showed that the main agronomic traits of the selected lines of the iR900 are essentially identical to the recurrent parent R900. The multi-site trials also showed that non-significant differences were found between the iS1000 hybrids and the original Super 1000. Given the potential trade-offs between resistance and yield, comprehensive traits should be considered when selecting final target plants after the molecular marker-based foreground and background selection (Nelson et al., 2018). The success of integrating phenotypic selection along with the background selection in the breeding programs for selecting superior recombinants has been demonstrated earlier by Ellur et al. (2016). The results in this study also indicate that a wise strategy to overcome linkage drag in breeding for resistance is to use commercial variety harboring resistance genes as a donor rather than the wild and landraces with the resistance genes. It is expected that favorable genes are accumulated in the improved donor and subsequently transfer of these genes would help in further improving the background of the pyramided lines (Pradhan et al., 2016).

## Conclusion

Significant resistance was achieved for overcoming BB and rice blast diseases in the improved parental R900 lines (iR900) and the corresponding hybrids, the improved Super 1000 hybrids (iS1000). In addition to improved disease resistance, field trials and large-scale demonstrations on multi-locations showed that iS1000 hybrids maintain super-high yield and exhibit identical agronomic traits to the original “Super 1000,” indicating a successful practice for trait improvement with genomics-assisted breeding. The present study also implies that it is feasible to conduct genetic improvement for other crops using a similar approach.

## Data Availability Statement

The original contributions presented in the study are included in the article/supplementary material, further inquiries can be directed to the corresponding authors.

## Author Contributions

ZH, JP, and YX designed the research. ZH, CW, ZXu, JC, CY, and YL drafted the manuscript. ZH, CW, HY, ZXu, JC, ZL, CY, HY, ZXi, NJ, JH, and YL performed the experiments and data analysis. ZH, JP, KZ, YX, ZXu, JC, CY, NJ, JX, BT, and YL revised and finalized the manuscript. All authors read and approved the final manuscript.

## Funding

This work was supported by the program Super Hybrid Rice Innovation Team, the National Natural Science Foundation of China (U20A2035, 32072412), the Central Public-interest Scientific Institution Basal Research Fund for Chinese Academy of Tropical Agricultural Sciences (No. 1630032019037), and the Hainan Yazhou Bay Seed Laboratory (B21HJ0218).

## Conflict of Interest

ZH, ZXu, JC, ZL, CY, HY, JX, BT, and JP were employed by Huazhi Bio-Tech Co., Ltd., Changsha, China. NJ was employed by Key Laboratory of Southern Rice Innovation and Improvement, Ministry of Agriculture and Rural Affairs, Hunan Engineering Laboratory of Disease and Pest Resistant Rice Breeding, Yuan Longping High-Tech Agriculture Company Ltd., Changsha, China. The remaining authors declare that the research was conducted in the absence of any commercial or financial relationships that could be construed as a potential conflict of interest.

## Publisher's Note

All claims expressed in this article are solely those of the authors and do not necessarily represent those of their affiliated organizations, or those of the publisher, the editors and the reviewers. Any product that may be evaluated in this article, or claim that may be made by its manufacturer, is not guaranteed or endorsed by the publisher.
